# Clinical Impacts of *Pseudomonas aeruginosa* Isolation in Patients with Bronchiectasis: Findings from KMBARC Registry

**DOI:** 10.3390/jcm13175011

**Published:** 2024-08-24

**Authors:** Jinhwa Song, Sooim Sin, Hye-Rin Kang, Yeon-Mok Oh, Ina Jeong

**Affiliations:** 1 Division of Pulmonary, Allergy and Critical Care Medicine, Department of Internal Medicine, Veterans Health Service Medical Center, Seoul 05368, Republic of Korea; jhsong1215@gmail.com (J.S.); mango8817@gmail.com (H.-R.K.); 2 Division of Pulmonary and Critical Care Medicine, Department of Internal Medicine, National Medical Center, Seoul 04564, Republic of Korea; sooim216@gmail.com; 3 Department of Pulmonary and Critical Care Medicine, Asan Medical Center, University of Ulsan College of Medicine, Seoul 05505, Republic of Korea

**Keywords:** bronchiectasis, *Pseudomonas aeruginosa*, prognosis

## Abstract

**Background:** *Pseudomonas aeruginosa* isolation in bronchiectasis is associated with a poor prognosis, including increased hospital admissions, exacerbation, and mortality. In this study, we aimed to evaluate the clinical characteristics and outcomes of *P. aeruginosa* isolation from patients with bronchiectasis in South Korea. **Methods:** This multicenter prospective cohort study analyzed 936 patients with bronchiectasis. We examined the prevalence of *P. aeruginosa* isolates and other microbiological characteristics. Additionally, the clinical characteristics related to disease severity and 1-year prognosis were compared between patients with and without *P. aeruginosa* isolation. Propensity score matching was used to mitigate confounding biases. **Results:** Of the 936 patients with bronchiectasis, *P. aeruginosa* was isolated from 89. A total of 445 matched patients—356 patients without (non-*Pseudomonas* group) and 89 with (*Pseudomonas* group) *P. aeruginosa* isolation—were analyzed. The *Pseudomonas* group showed poorer lung function, greater involvement of radiographic bronchiectasis, and a higher proportion of cystic bronchiectasis than the non-*Pseudomonas* group. After one year, more patients in the *Pseudomonas* group were admitted for bronchiectasis than in the non-*Pseudomonas* group. Moreover, the Bronchiectasis Health Questionnaire scores were significantly lower in the *Pseudomonas* group than in the non-*Pseudomonas* group. **Conclusions:** The isolation of *P. aeruginosa* was independently associated with increased disease severity and poor clinical outcomes in Korean patients with bronchiectasis.

## 1. Introduction

Non-cystic fibrosis bronchiectasis (henceforth referred to as bronchiectasis) poses a significant medical challenge owing to its chronic and progressive nature. Bronchiectasis significantly impairs patient quality of life and imposes a substantial disease burden due to recurrent exacerbations and infections [1]. Despite its prevalence, bronchiectasis is often overlooked, likely due to the uncertainty regarding its treatment and etiology [2].

Amidst the uncertainty regarding bronchiectasis, one clear fact has emerged: *Pseudomonas aeruginosa* is the most important pathogenic agent [3,4]. Playing a pivotal role in pathogenesis and prognosis, *P. aeruginosa* activates inflammation and promotes tissue damage in the airways through the secretion of proteins and cellular components, which is a crucial mechanism in bronchiectasis development [5,6]. Recognized as a key prognostic factor, its colonization in patients with bronchiectasis is associated with adverse clinical outcomes, including frequent pulmonary infections, hospital admissions, declining pulmonary function, and high mortality rates [7,8,9,10]. Microbiome research supports these findings, highlighting the impact of *P. aeruginosa*-dominated microbiomes on lung function decline and frequent exacerbations compared with those associated with other microbiome profiles [11].

The incidence of *P. aeruginosa* colonization in bronchiectasis varies across geographical regions and ethnic populations, ranging from 9% to 33% [9,12,13]. Although studies in Asian countries report an incidence of 25–30%, there is limited information on *P. aeruginosa* colonization in bronchiectasis in South Korea. In addition, few studies have explored the clinical implications of *P. aeruginosa* colonization, owing to the lack of large bronchiectasis cohorts. This study aimed to address this gap by investigating the incidence and clinical impact of *P. aeruginosa* colonization in patients with bronchiectasis in South Korea.

## 2. Materials and Methods

### 2.1. Study Population

We analyzed the data from the Korean Multicenter Bronchiectasis Audit and Research Collaboration (KMBARC) registry [14,15]. The KMBARC registry is a prospective cohort study conducted across more than 26 centers in South Korea. Participants were enrolled from August 2018 to April 2021 (trial No. KCT 0003088). KMBARC adhered to the protocol of the European Multicenter Bronchiectasis Audit and Research Collaboration (EMBARC) [16] to gather clinical information from patients with bronchiectasis in South Korea. The data collected included bronchiectasis health questionnaires [17], assessments of fatigue and depression assessments, blood test results, exacerbation definitions, and details of emergency room or hospital visits. 

The inclusion criteria for this study were as follows: (1) patients aged 18 years or older with or without respiratory symptoms (cough, sputum, or respiratory infection) and bronchiectasis involving one or more lobes confirmed by chest computed tomography; (2) patients hospitalized for respiratory disease in a stable condition at the time of enrollment and at least 4 weeks post-discharge. The exclusion criteria were as follows: (1) cystic fibrosis; (2) traction bronchiectasis observed in interstitial lung disease; (3) active treatment for pneumonia, pulmonary tuberculosis (TB), or non-tuberculous mycobacterial pulmonary disease; (4) inability or unwillingness to provide informed consent; (5) pregnancy. Informed consent was obtained from all the participants. The Institutional Review Board of the National Medical Center of Korea approved the study design (IRB no. H-1807-092-015).

### 2.2. Data Collection

Epidemiological data, including age, sex, body mass index (BMI), number of bronchiectasis diagnoses, smoking history, total smoking history, and comorbidities (chronic obstructive pulmonary disease [COPD], asthma, history of TB, rhinosinusitis, cardiovascular disease, pulmonary hypertension, stroke, diabetes mellitus, osteoporosis, chronic kidney disease, malignancy, depression, and anxiety) were examined. Clinical details included radiographic involvement; medication history, including the use of inhalers and oral antibiotics; microbiology and source of sputum; pulmonary function tests; Modified Medical Research Council (mMRC) dyspnea scale; sputum volume; presence of hemoptysis; exacerbations; hospitalization within the previous year of enrollment. Indicators of clinical severity included the Bronchiectasis Health Questionnaire (BHQ), bronchiectasis severity index (BSI) [18], FACED score (forced expiratory volume in 1 s (FEV_1_), age, chronic colonization by *Pseudomonas aeruginosa*, radiological extension, and dyspnea) [19]. The clinical and epidemiological characteristics of the patients enrolled in the KMBARC registry were reported by Lee et al. [15].

### 2.3. Definition of Terminology and Outcomes

The etiology of bronchiectasis was determined by the attending pulmonologist through interviews and clinical assessment of the patient.

Exacerbation of bronchiectasis was defined according to the international consensus [20] and characterized by a deterioration of three or more of the following six symptoms that persisted for 48 h or more and improved with treatment: (1) coughing, (2) sputum volume and/or consistency, (3) sputum purulence, (4) dyspnea and/or exercise tolerance, (5) fatigue and/or malaise, and (6) hemoptysis.

*Pseudomonas* colonization was defined as the identification of *Pseudomonas* in at least two separate sputum culture studies with the following criteria: (1) *Pseudomonas* was isolated from a sputum sample obtained during stable status, with a history of *Pseudomonas* isolation; (2) *Pseudomonas* was isolated from the sputum culture at enrollment and at the 1-year follow-up sputum culture; (3) a history of *Pseudomonas* isolation and *Pseudomonas* was isolated in the 1-year follow-up sputum culture. 

The primary outcome was the occurrence of exacerbations at a 1-year follow-up. Secondary outcomes included hospitalization for bronchiectasis, hemoptysis requiring hospitalization, antibiotic use, changes in clinical severity indices (BHQ, BSI, and FACED scores), and changes in lung function at a 1-year follow-up.

### 2.4. Statistical Analysis 

For patients colonized with *Pseudomonas*, propensity score matching was conducted by adjusting for age and sex at a ratio of 1:4 in the control group. The MatchIt package in R was used for analysis. 

Categorical variables were compared using the chi-square or Fisher’s exact test, and continuous functions were compared using Student’s *t*-test or Wilcoxon rank-sum analysis. Logistic and negative binomial regression analyses were performed to assess the risks of hemoptysis, exacerbation, and hospital admission during the 1-year follow-up period. Multivariate regression analyses were adjusted for age, sex, and BMI. R software (version 4.3.1; The R Foundation for Computing, Vienna, Austria) and STATA (version 17; StataCorp, College Station, TX, USA) were used for statistical analyses.

## 3. Results

### 3.1. Baseline Characteristics of the Population

**Figure 1:**
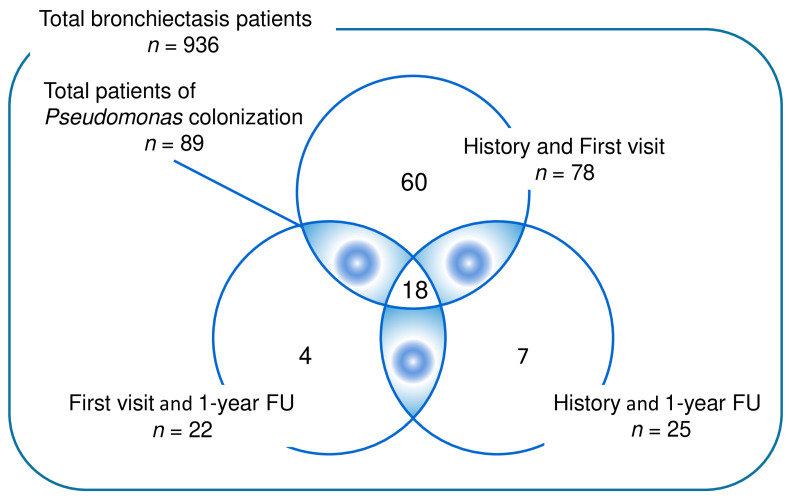
Venn diagram representing the isolation of *Pseudomonas aeruginosa* from the KMBARC registry. Abbreviations: History, History of *Pseudomonas aeruginosa* isolation from respiratory secretion; First visit, *Pseudomonas aeruginosa* isolation from respiratory secretion at first visit; 1-year FU, *Pseudomonas aeruginosa* isolation from respiratory secretion at the 1-year follow-up.

Of the 936 patients enrolled in KMBARC, 89 with *Pseudomonas* colonization were identified (Figure 1). The mean age of the total population was 64.4 ± 9.4 years (Table 1). The duration of bronchiectasis was significantly longer in the *Pseudomonas* colonization group than in the non-colonization group (with mean ± standard deviation in years of 3.7 ± 1.7 vs. 3.2 ± 1.6, *p* = 0.006). Additionally, the proportion of patients with COPD was significantly higher in the *Pseudomonas* group (43.8% vs. 32.0%; *p* = 0.036) (Table 1). Radiologic extension of bronchiectasis was greater in the *Pseudomonas* group, affecting more lobes on average (2.7 ± 1.5 vs. 2.3 ± 1.6, *p* = 0.021), particularly the lingula and the left lower lobe (Table 1).

Of the 325 patients who provided samples at stable status, microorganisms were isolated from 151 (46.5%) samples. *P. aeruginosa* was the most frequently isolated microorganism, found in 92/151 (60.9%) of the positive cultures (Table 2). Other less commonly isolated organisms included *Streptococcus viridans* (13.3%), *Staphylococcus aureus* (6.6%), *Klebsiella pneumoniae* (6.0%), *Hemophilus influenza* (4.6%), *Escherichia coli* (4.6%), *Enterobacter cloacae* (2.7%), *Streptococcus pneumonia* (2.0%), and *Moraxella catarrhalis* (1.3%) (Table 2). 

### 3.2. Clinical Characteristics Related to Bronchiectasis

The *Pseudomonas* group had significantly poorer lung function across all measures related to bronchiectasis than the non-*Pseudomonas* group, with lower pre- and post-bronchodilator forced expiratory volume in one second (FEV_1_) and FVC both in absolute terms and as a percentage of the predicted values (*p* < 0.001) (Table 3). However, there was no significant difference in the respiratory symptoms or acute exacerbation history between the two groups. The *Pseudomonas* group had a higher FACED score (3.1 ± 1.5 vs. 1.9 ± 1.6) and BSI score (10.5 ± 3.4 vs. 6.6 ± 3.6), indicating more severe disease, with *p* < 0.001. The BHQ score, which reflects health-related quality of life, was also significantly lower in the *Pseudomonas* group (10.5 ± 3.4 vs. 6.6 ± 3.6, *p* = 0.018) (Table 3).

### 3.3. Clinical Outcomes Based on Pseudomonas Colonization

Over a 1-year follow-up period, the *Pseudomonas* group showed a higher risk of hospitalization owing to bronchiectasis (adjusted odds ratio [aOR] 2.19 (95% confidence interval [CI] 1.08–4.42)) and a greater need for antibiotic treatment (aOR 3.04 (95% CI 1.17–7.91)). Additionally, the *Pseudomonas* group had a lower BHQ score (60.5 ± 11.7 vs. 67.7 ± 11.2, *p* < 0.001), indicating poorer quality of life (Table 4).

## 4. Discussion

In this multicenter cohort study of 936 patients, 89 patients with *Pseudomonas* colonization exhibited distinctive clinical characteristics and outcomes compared with those of the control group. The presence of *Pseudomonas* colonization significantly correlated with poorer lung function, higher prevalence of COPD, prolonged bronchiectasis duration, and increased radiological extension, notably affecting the lingula and left lower lobe. Furthermore, patients with *Pseudomonas* colonization had an elevated risk of hospitalization, increased antibiotic requirements over one year, and a lower BHQ score after one year, indicating a diminished quality of life among patients with bronchiectasis. Importantly, these findings remained robust even after adjusting for demographic factors, highlighting the impact of *Pseudomonas* colonization on the clinical trajectory of bronchiectasis.

*P. aeruginosa* is a frequently occurring pathogen in patients with bronchiectasis and has detrimental effects on disease progression [21]. Research has shown that its isolation is associated with worse respiratory symptoms [9], poor quality of life [22], and increased airflow obstruction [23]. Furthermore, colonization by *P. aeruginosa* has been associated with a rapid decline in lung function [7] and frequent exacerbation of bronchiectasis with cystic fibrosis [24] and non-cystic fibrosis bronchiectasis [10,11,25]. It significantly affects the progression and severity of bronchiectasis, resulting in poor lung function, severe radiological features [3,9,18,26], frequent hospitalizations [2,8], and exacerbations [18]. Our study showed similar results for hospital admission and acute exacerbation; no significant difference was observed in acute exacerbation.

In patients with bronchiectasis, those with *P. aeruginosa* infection experience a faster annual decline in FEV_1_ [27], especially older patients and those with chronic infection [7]. Therefore, *P. aeruginosa* infection is associated with a rapid decline in lung function, leading to poor respiratory health and ultimately contributing to disease progression [28]. Various risk factors have been identified for *P. aeruginosa* infection in patients with bronchiectasis. These include increased duration of bronchiectasis, use of proton pump inhibitors (PPIs) [29], cystic and extensive bronchiectasis, and severe radiological involvement [29,30]. Similar to the results of a previous study, we found that the FEV_1_ and FVC were lower in Korean patients with bronchiectasis and *Pseudomonas* colonization. However, we did not observe any longitudinal changes owing to the short follow-up period. In the EMBARC study, 25.5% of the cohort had COPD and 31% had asthma, whereas in this study, 34.4% and 23% of the patients had COPD and asthma, respectively. This difference in the airway disease proportion may have led to differences in pharmacological therapy, which in turn affects lung function and disease prognosis [31].

The BHQ is a simple tool that can be used to assess the quality of life of patients with bronchiectasis and has been validated in many countries [17,32], including Korea [33]. This questionnaire evaluates fatigue, performance, emotional states, and respiratory symptoms. It has a high predictive value for lung function and the risk of severe exacerbation [33]. To our knowledge, this study is the first to demonstrate a significant association between lower BHQ scores in patients with *Pseudomonas* infection and their persistence after a follow-up period of 1 year.

Previous studies have reported varying rates of *Pseudomonas* colonization in patients with non-cystic fibrosis bronchiectasis, ranging from 8% to 33% [12,21,29], with a typical incidence of 25%. However, in our study, we observed a very low prevalence of 9.5%. The frequency of colonization can be affected by various factors such as colonization definition, timing of follow-up, country, and race of the study participants [8,34,35]. In our study, the results were based on two separate specimen identifications taken one year apart. It is important to note that the typical definition of colonization requires two positive cultures that are at least three months apart for over a year. Therefore, the true colonization rate may have been underestimated.

The strength of this study lies in its multicenter prospective cohort design conducted within an Asian population, encompassing a large participant pool. Unlike many studies that involved only a limited number of patients recruited from a single center, our investigation has a broader scope. To the best of our knowledge, this is the first prospective study to demonstrate the frequency of acute exacerbations in an Asian population. This is the first study to prospectively demonstrate the impact of *P. aeruginosa* colonization on the quality of life of patients with bronchiectasis, as measured using the BHQ score. 

This study has several limitations. First, the 1-year follow-up period may have been insufficient for a comprehensive assessment of changes in lung function and the frequency of acute exacerbations. Five years of data were not available because the data processing was not completed. Second, we could not establish causality between the adverse outcomes observed in patients with *P. aeruginosa* colonization and their poorer baseline characteristics, including compromised lung function and more extensive bronchiectasis, than those in patients without *P. aeruginosa* colonization. To address this limitation, we used propensity score matching and adjusted for baseline factors. Furthermore, the prospective nature of our study helps to mitigate this limitation. Further prospective studies with long-term follow-ups are required. Finally, because COVID-19 occurred during the follow-up period of this cohort, the decrease in the number of follow-up patients may have been influenced by the reluctance to visit the hospital due to COVID-19 concerns or the inability of patients affected by COVID-19 to attend their appointments. This may have resulted in a loss of data after one year, potentially introducing bias.

## 5. Conclusions

In our multicenter prospective cohort study, patients with bronchiectasis and *P. aeruginosa* colonization exhibited worse lung function, prolonged duration of bronchiectasis, and greater radiological extension. They experienced an elevated risk of hospitalization and increased antibiotic use during follow-up, as evidenced by low BHQ scores. Further studies are needed to observe the long-term outcomes in patients with bronchiectasis and *P. aeruginosa* colonization.

## Figures and Tables

**Table 1:** Baseline Characteristics of matched patients.

Variables	Total *n* = 445	*Pseudomonas* Group *n* = 89	Non-*Pseudomonas* Group *n* = 356	*p*-Value
Age	64.4 ± 9.4	63.5 ± 9.8	64.6 ± 9.3	0.358
Sex, Female, n (%)	289 (64.9)	56 (62.9)	233 (65.5)	0.655
BMI	22.9 ± 3.5	23.0 ± 3.5	23.0 ± 3.5	0.120
Median duration between BE diagnosed to enrollment, (IQR)	3 (1–6)	3 (1–6)	3 (1–6)	0.011
Smoking history, n (%)				0.469
Current smoker	14 (3.2)	1 (1.1)	13 (3.7)	
Former smoker	111 (25.1)	23 (25.8)	88 (24.9)	
Never smoker	318 (71.8)	65 (73.0)	253 (71.5)	
Pack-years	3.2 (1.3)	2.9 (1.2)	3.3 (1.3)	0.233
Past medical history, n (%)				
COPD	152 (34.4)	39 (43.8)	113 (32.0)	0.036
Asthma	102 (23.0)	14 (15.7)	88 (24.9)	0.069
History of TB	148 (36.1)	35 (39.3)	123 (35.2)	0.474
Rhinosinusitis	28 (6.3)	9 (10.1)	19 (5.4)	0.100
Cardiovascular disease	131 (29.4)	24 (27.0)	107 (30.1)	0.567
Pulmonary hypertension	15 (3.5)	1 (1.2)	14 (4.1)	0.323
Stroke	11 (2.6)	1 (1.2)	10 (2.9)	0.700
Diabetes mellitus	58 (13.2)	5 (6.8)	52 (14.7)	0.049
Osteoporosis	48 (10.8)6	6 (6.7)	42 (11.8)	0.167
Chronic renal failure	9 (2.1)	0 (0.0)	9 (2.6)	0.131
Cancer, total	40 (9.0)	4 (4.5)	36 (10.2)	0.095
Depression	19 (4.3)	2 (2.3)	17 (4.8)	0.390
Anxiety	13 (2.9)	2 (2.3)	11 (3.1)	0.999
Radiologic severity of the bronchiectasis				
Total number of involved lobes, mean (SD)	2.4 (1.6)	2.7 (1.5)	2.3 (1.6)	0.021
Patients with relevant lobe involvement, n (%)				
RUL	175 (39.3)	43 (48.3)	132 (37.1)	0.052
RML	257 (57.8)	53 (59.6)	204 (57.3)	0.701
RLL	248 (55.7)	57 (64.0)	191 (53.7)	0.077
LUL	159 (35.7)	33 (37.1)	126 (35.4)	0.767
Lingula	224 (50.3)	57 (64.0)	167 (46.9)	0.004
LLL	319 (71.7)	74 (83.2)	245 (68.8)	0.007
Cystic bronchiectasis, n (%)	200 (44.9)	53 (59.6)	147 (41.3)	0.002
Laboratory findings				
WBC (10^3^/uL)	9.6 (3.9) (n = 65)	10.3 (3.2) (n = 14)	9.4 (4.0) (n = 51)	0.437
Neutrophil (%)	70.5 (10.8) (n = 68)	73.3 (7.2) (n = 16)	69.7 (11.6) (n = 52)	0.146
Lymphocyte (%)	20.8 (9.4) (n = 68)	18.4 (6.4) (n = 16)	21.6 (10.1) (n = 52)	0.141
Monocyte (%)	6.3 (2.1) (n = 68)	6.6 (1.3) (n = 16)	6.2 (2.3) (n = 52)	0.454
Eosinophil (%)	1.5 (1.2) (n = 68)	1.0 (1.1) (n = 16)	1.6(1.3) (n = 52)	0.117
Hemoglobin (g/dL)	12.6 (1.5) (n = 65)	12.9 (2.0) (n = 14)	12.5(1.4) (n = 51)	0.456
Platelet (10^3^/uL)	283.1(83.0) (n = 65)	303.1(106.3) (n = 14)	277.6(75.8) (n = 51)	0.314
Total bilirubin (mg/dL)	0.50 (0.03) (n = 70)	0.50 (0.05) (n = 16)	0.5 (0.04) (n = 54)	0.946
Serum albumin (g/dL)	4.0(0.5) (n = 46)	4.1 (0.5) (n = 10)	4.0 (0.5) (n = 36)	0.665
BUN (mg/dL)	15.2 (5.8) (n = 70)	14.4 (3.8) (n = 16)	15.4 (6.3) (n = 54)	0.532
Creatinine (mt/dL)	0.75 (0.24) (n = 70)	0.80 (0.19) (n = 16)	0.73(0.25) (n = 54)	0.318
Past medication history				
Long term macrolide use (≥1 month)	20 (6.7)	8 (11.3)	12 (5.3)	0.081
Inhaled corticosteroid use *	78 (17.5)	11 (12.4)	67 (18.8)	0.152
Long term use of bronchodilators †	218 (66.1)	51 (71.8)	167 (64.5)	0.246

BE, Bronchiectasis; BMI, Body Mass Index; BUN, Blood Urea Nitrogen; COPD, Chronic Obstructive Pulmonary Disease; IQR, Interquartile Range; LLL, Left Lower Lobe; LUL, Left Upper Lobe; RLL—Right Lower Lobe; RML—Right Middle Lobe; RUL, Right Upper Lobe; SD, Standard Deviation; TB, Tuberculosis * including inhaled corticosteroid (ICS) and ICS/long-acting beta-agonist (LABA) † including ICS/LABA, LABA, and LABA/long-acting muscarinic antagonist. Feature of microbiologic isolations.

**Table 2:** Isolated microbiology of the study population.

Variables	Patients Obtained Microbiologic Samples from the Airway in Medically Stable Status *n* = 341
Patients from whose respiratory secretion isolated microorganism, n (%) *	159 (46.6)
Microorganism, n (%) ^#^	
*Pseudomonas aeruginosa*	92/159 (57.9)
*Streptococcus viridans*	23/159 (14.5)
*Klebsiella pneumonia*	10/159 (6.3)
*Staphylococcus aureus*	10/159 (6.3)
*Hemophilus influenzae*	7/159 (4.4)
*Escherichia coli*	7/159 (4.4)
*Enterobacter cloacae*	4/159 (2.5)
*Streptococcus pneumonia*	3/159 (1.9)
*Moraxella catarrhalis*	2/159 (1.2)
*Acinetobacter baumannii*	1/159 (0.6)
Others ^§^	2/159 (1.2)
Source of microbiology. n (%) ^†^	
Sputum	269 (95.7%)
Induced sputum	10 (2.6%)

BAL, bronchoalveolar lavage * In the non-pseudomonas group, 104 did not perform a sputum test. Among them, 12 patients refused to be tested, 6 were unable to expectorate sputum due to lack of sputum, and 86 had no reason listed. ^#^ The denominators were patients with positive sputum culture results. ^§^ Others include *S. maltophilia, pasteurella multocida* ^†^ The denominators were number of microbiology-detected specimen.

**Table 3:** Characteristics related to bronchiectasis severity according to *Pseudomonas* groups.

	Total *n* = 445	*Pseudomonas* Group *n* = 89	Non-*Pseudomonas* Group *n* = 356	*p*-Value
Lung function, mean (SD)				
Pre BD FEV_1_, absolute [L]	1.59 (0.58)	1.35 (0.49)	1.66 (0.59)	<0.001
Pre BD FEV_1_, % predicted	64.2 (19.0)	56.5 (18.1)	66.0 (18.8)	<0.001
Pre BD FVC, absolute [L]	2.51 (0.76)	2.24 (0.72)	2.57 (0.75)	<0.001
Pre BD FVC, % predicted	73.2 (15.7)	65.7 (15.5)	74.8 (15.3)	<0.001
Post BD FEV_1_, absolute [L]	1.71 (0.58)	1.50 (0.57)	1.76 (0.58)	0.001
Post BD FEV_1_, % predicted	66.6 (18.8)	58.5 (17.6)	68.5 (18.6)	<0.001
Post BD FVC, absolute [L]	2.55 (0.75)	2.26 (0.72)	2.61 (0.75)	<0.001
Post BD FVC, % predicted	73.9 (15.4)	66.3 (15.0)	75.6 (15.0)	<0.001
Respiratory symptom				
mMRC, mean (SD)	1.00 (0.84)	1.12 (0.99)	0.07 (0.80)	0.165
Patient with each mMRC, n (%)				0.175
mMRC grade ≥ 2	84 (19.0)	22 (24.7)	62 (17.5)	0.121
mMRC grade ≥ 3	28 (6.3)	9 (10.1)	19 (5.4)	0.100
mMRC grade 4	5 (1.1)	3 (3.4)	2 (0.56)	0.058
Sputum color				0.242
Mucus	283 (65.2)	59 (66.3)	224 (64.9)	
Mucopurulent	112 (25.8)	18 (20.2)	94 (27.3)	
Purulent	34 (7.8)	10 (11.2)	24 (7.0)	
Very Purulent	5 (1.2)	2 (2.3)	3 (0.9)	
Sputum volume, cc/day mean (SD)	26.2 (25.0)	21.0 (22.3)	29.2 (26.1)	0.145
h/o major hemoptysis, n (%)	29 (10.3)	9 (14.1)	20 (9.1)	0.253
Acute exacerbation history				
Presence of AE, n (%)	209 (54.3)	46 (54.8)	163 (54.2)	0.921
Number of AE, mean (SD)	1.5 (2.3)	1.6 (2.2)	1.5 (2.4)	0.794
More than 2 annual exacerbations, n (%)	129 (33.5)	30 (35.7)	99 (32.9)	0.628
Requiring antibiotics, mean (SD)	1.7 (2.1)	2.3 (2.5)	1.5 (2.0)	0.096
Requiring ER visit, n (%)	31 (7.0)	9 (10.1)	22 (6.2)	0.198
Requiring hospital admission, n (%)	81 (18.3)	18 (20.2)	63 (17.8)	0.596
Hospital admission due to bronchiectasis, n (%)	118 (26.6)	30 (33.7)	88 (24.9)	0.091
BHQ score, mean (SD)	63.7 (10.7)	61.3 (11.0)	64.3 (10.6)	0.018
FACED score, mean (SD)	2.1 (1.7)	3.1 (1.6)	1.9 (1.6)	<0.001
BSI score, mean (SD)	7.4 (3.9)	10.5 (3.4)	6.6 (3.6)	<0.001

AE, Acute exacerbation; BD, Bronchodilator; BHQ, Bronchiectasis Health Questionnaire; BSI, Bronchiectasis Severity Index, ER, Emergency room; FACED, a scoring system for assessing the severity of bronchiectasis including the components FEV_1_, age, chronic colonization, extension, and dyspnea; FEV_1_, Forced Expiratory Volume in one second; FVC, Forced Vital Capacity; mMRC, Modified Medical Research Council Dyspnea Scale; SD, Standard Deviation.

**Table 4:** Clinical outcomes of the study population after 1 year.

	Total *n* = 445	*Pseudomonas* Group *n* = 89	Non-*Pseudomonas* Group *n* = 356	*p*-Value
Risk of exacerbation, crude odds ratio (CI)		1.78 (1.07–2.97)	Ref	0.027
Risk of exacerbation, adjusted odds ratio (CI) *		1.66 (0.98–2.82)	Ref	0.058
Risk of exacerbation of ED visit, crude odds ratio (CI)		1.95 (0.58–6.57)	Ref	0.283
Risk of exacerbation of ED visit, adjusted odds ratio (CI) *		1.64 (0.46–5.83)	Ref	0.441
Risk of admission due to bronchiectasis, crude odds ratio (CI)		2.29 (1.18–4.43)	Ref	0.014
Risk of admission due to bronchiectasis, adjusted adds ratio (CI) *		2.19 (1.08–4.42)	Ref	0.028
Risk of hemoptysis, crude odds ratio (CI)		1.48 (0.82–2.64)	Ref	0.191
Risk of hemoptysis, adjusted adds ratio (CI) *		1.54 (0.85–2.81)	Ref	0.153
Risk of antibiotics treatment, crude odds ratio (CI)		3.08 (1.22–7.80)	Ref	0.018
Risk of antibiotics treatment, adjusted adds ratio (CI) *		3.04 (1.17–7.91)	Ref	0.022
				
BHQ score *	66.0 (11.7) (n = 236)	60.5 (11.7) (n = 54)	67.7 (11.2) (n = 182)	<0.001
FACED score *	2.1 (1.6) (n = 37)	3.0 (2.2) (n = 8)	1.9 (1.4) (n = 29)	0.080
BSI score *	7.6 (4.3) (n = 37)	8.8 (4.9) (n = 8)	7.2 (4.1) (n = 29)	0.345

BHQ, Bronchiectasis Health Questionnaire; BSI, Bronchiectasis Severity Index; ED, emergency department, FACED, a scoring system for assessing the severity of bronchiectasis, including FEV_1_, age, chronic colonization, extension, and dyspnea; CI, confidence interval. * Adjusted for age, sex, and BMI.

## Data Availability

Data supporting the conclusions of this article are available from the corresponding author upon request.

## Abbreviations

*P. aeruginosa*—*Pseudomonas aeruginosa*; FACED—F: forced expiratory volume in 1 s [FEV_1_]; A: age; C: chronic colonization by *Pseudomonas aeruginosa*; E: radiological extension [number of pulmonary lobes affected]; D: dyspnea; BSI—Bronchiectasis Severity Index; BHQ—Bronchiectasis Health Questionnaire.
